# Spreading of Alu Methylation to the Promoter of the *MLH1* Gene in Gastrointestinal Cancer

**DOI:** 10.1371/journal.pone.0025913

**Published:** 2011-10-12

**Authors:** Xiyin Wang, Jing Fan, Dong Liu, Siqing Fu, Sigurdur Ingvarsson, Huiping Chen

**Affiliations:** 1 Department of Medical Genetics, Tongji Medical College, Huazhong University of Science and Technology, Wuhan, Hubei, China; 2 Institute for Experimental Pathology and Faculty of Medicine, University of Iceland, Keldur, Reykjavik, Iceland; University of Barcelona, Spain

## Abstract

The highly repetitive Alu retroelements are regarded as methylation centres in the genome. Methylation in the gene promoters could be spreading from them. Promoter methylation of *MLH1* is frequently detected in cancers, but the underlying mechanism is unclear. The aim of this study is to understand whether the methylation in the Alu elements is associated with promoter methylation in the MLH1 gene. Bisulfite genomic sequencing was used to analyse the CpG sites of the 5′ end (promoter, exon 1 and Alu-containing intron 1) of the *MLH1* gene in colorectal cancer cells and tissues, and gastric cancer tissues. Hypomethylation in the Alu elements and hypermethylation in the promoters and the regions between the promoters and the Alu elements were detected in two cancer cell lines and seven cancer tissues. However, demethylation or hypomethylation of the MLH1 promoter and regions between promoter and the Alu elements, and hypermethylation in the Alu elements, were identified in the normal tissues. *MLH1* promoter methylation may spread from Alu elements that are located in intron 1 of the *MLH1* gene. The *trans-acting* elements binding to the mutation sites could play a role in the methylation spreading.

## Introduction

*MLH1* is a major mismatch repair gene which plays a role in maintaining the stability of the genome. *MLH1* dysfunction may cause a high rate of gene mutations in the genome. Promoter methylation of *MLH1*, especially the C region (−310 to −240, relative to the initiation codon) containing 8 CpG sites, is a frequent event in cancer, which could result in loss of *MLH1* expression [1]–[3]. To date, the mechanism of *MLH1* methylation is unclear. Our previous studies have shown that *MLH1* methylation may be associated with *MLH1* -93SNP [4], [5]. However, the molecular basis behind this is unknown.

Alu is one of the repetitive elements in the genome, which is hypermethylated in normal cells [6]. Alu elements are believed to be methylation centres in the genome [7]. In cancer, gene promoter methylation may spread from adjacent repetitive elements [7]. Graff et al. [8] mapped the methylation patterns of *E-cadherin* and *von Hippel-Lindau* tumour suppressor genes in both normal and neoplastic cells, and found that boundaries exist between the unmethylated promoters and the nearby hypermethylated Alu elements, to maintain the unmethylated status of the promoters in normal cells, and that the boundaries may be progressively overriden by methylation of the Alu elements, resulting in promoter methylation in neoplasia.

**Figure 1:**
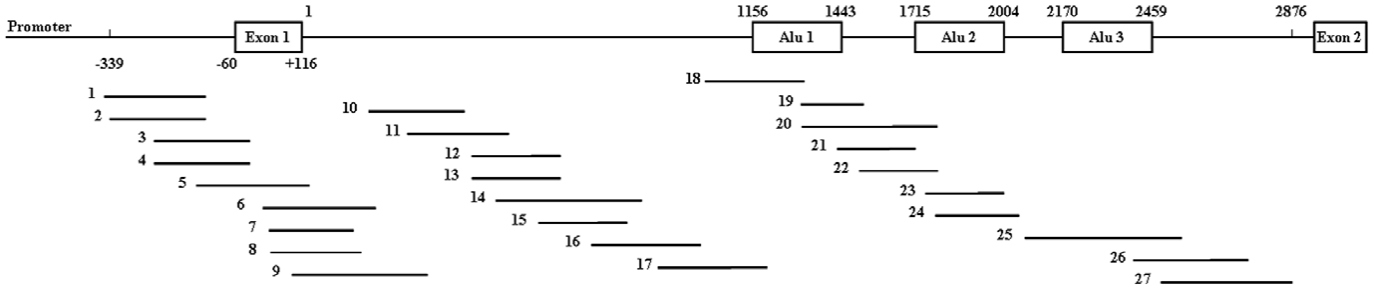
A graphic overview of the 5′ end (promoter, exon 1, intron 1 and exon 2) of the human *MLH1* gene and 27 amplicons in relation to it. There are three Alu elements in intron 1, whose sizes and locations are shown by nucleotide numbers. The relative sizes and locations of the 27 PCR amplicons are depicted by bold lines, which cover a region from −339 to 116+2876.

Three Alu elements have been identified in intron 1 of *MLH1* by searching a database of human Alu repeat elements (Fig. 1). No Alu elements exist in the *MLH1* promoter region. The methylation status of each CpG site within Alu elements of *MLH1* has not been pinpointed. It is possible that *MLH1* promoter methylation occurs from nearby Alu elements. To test this, we analysed the methylation status of all CpG sites of the *MLH1* 5′ end (C region containing promoter, exon 1 and majority of intron 1) (Fig. 1) in colorectal cancer cells and tissues, gastric cancer tissues and normal tissues using bisulfite genomic sequencing, and found that Alu elements in the intron 1 of *MLH1* are hypomethylated, and the promoters and the regions between *MLH1* promoters and Alu elements are hypermethylated in cancers. However, in the normal tissues Alu elements are hypermethylated, and the promoters and the regions between promoters and Alu elements are not methylated or hypomethylated.

## Materials and Methods

### Ethics statement

This research has been approved by the review board of Huazhong University of Science and Technology. We obtained tissue samples with written informed consent from the participants involved in the study. The ethics committee specifically approved the procedures.

### Normal and cancer samples

Two colorectal cancer cell lines, RKO and SW48, with *MLH1* promoter methylation [2] were ordered from the Chinese Academy of Sciences (Shanghai, China). A total of 188 colorectal and 27 gastric cancer tissues with matched normal mucosa were obtained from Tongji hospital (Wuhan, China). The peripheral blood of a healthy individual was also obtained from Tongji hospital (Wuhan, China).

### Cell culture and DNA isolation

Cells were grown in DMEM supplemented with 10% foetal bovine serum at 37°C with 5% CO_2_ atmosphere. DNA was extracted from cells, cancer tissues, normal mucosa and blood samples using DNA isolation kit (Sangon, Shanghai, China) and TIANamp genomic DNA kit (Tiangen, Beijing, China).

### Characterisation of tumours

All tumours were assessed for microsatellite instability (MSI) using five microsatellite repeats (BAT25, BAT26, D2S123, D5S346, and D17S250), as described previously [1]. For the MSI positive tumours, the extracted DNA was converted using EZ DNA Methylation Kit (Zymo Research Corporation, Orange, CA, USA). *MLH1* methylation at cytosines at −250 and −252 relative to the initiation codon was assessed using combined bisulfite restriction analysis (COBRA) with BstUI [1].

### Immunohistochemical (IHC) staining

IHC staining for MLH1 proteins was performed on 5-µm sections from paraffin-embedded tumour and adjacent normal tissue blocks with antibody MLH1 (ab92312, abcam, UK). The sections were deparaffinised, rehydrated, and rinsed in tap water before antigen retrieval by boiling in a 0.01 M citrate buffer (pH 6.0) twice for 5 min. Sections were incubated with antibodies overnight at 4°C. IHC staining was visualised using the Strep ABC Complex/horseradish peroxidise (HPR). Tumours were graded by intensity of staining as negative, weakly positive, moderately positive and strongly positive.

### Bisulfite genomic sequencing

For COBRA-positive samples, the methylation status of *MLH1* 5′end was determined using bisulfite genomic sequencing. In total there were 27 PCR amplicons designed to cover the region from −339 to 116+2876, relative to the translation start site (Fig. 1). The primers used are shown in Table S1. PCR was performed at 95°C for 5 min followed by 40 cycles of 95°C for 30 sec, 55–61°C for 45 sec and 72°C for 1 min with a final extension at 72°C for 7 min. A hot start was used by adding the enzyme during the first cycle at about 72°C, after a preincubation time of 5 minutes at 95°C. The PCR products were tested in 2% agarose gel and then cloned into the pEASY-T1 vector (TransGen Biotech, Beijing, China). The colony PCR was undertaken to screen the positive colonies. The clones with the right sizes of PCR products were sequenced on an ABI sequencer with dye terminators (Applied Biosystems, Foster City, CA, USA). With sequencing results of five clones, the methylation frequency was determined for each CpG site.

### Mutation screening

The *MLH1* 5′ end for methylation analysis was sequenced using the unconverted DNA from cancer cells and tissues. The 10 pairs of primers used are shown in Table 1. PCR was performed at 95°C for 5 min followed by 35 cycles of 95°C for 30 sec, 58–66°C for 30 sec and 72°C for 1 min with a final extension at 72°C for 7 min. Then PCR products were directly sequenced using the ABI sequencer.

**Table 1:** Oligonucleotide sequences of the primers for mutation analysis.

Primer name	Primer sequence (5′-3′)	Genomic position	Product size (bp)
A	ACCTCAGCAGAGGCACACA	−369 to −351	393
	AATAACCCCTGCCACGAAC	+6 to +24	
B	CGTTTCCTTGGCTCTTCTGG	−27 to −7	343
	GGGGAGAGCGGTAAAGAAAC	IVS*1+181 to IVS1+200	
C	GTCAGGCCTTCTCCTTTTCC	IVS1+150 to IVS1+169	354
	AAAGTGCATCAGCCTGTCCT	IVS1+484 to IVS1+503	
D	ATTCATTTTGAGTTTCTTTCAAAAC	IVS1+442 to IVS1+466	381
	CCTACCACTCCAAACTGAAGC	IVS1+802 to IVS1+822	
E	TGACGTCCGTACGTTAATAGAAAA	IVS1+772 to IVS1+795	344
	CAAGCCACCAAGCTAGTATGTTT	IVS1+1093 to IVS1+1115	
F	TTGTACTGTGCCAGAATACTGTAAA	IVS1+1060 to IVS1+1084	365
	TGTAATCCCAGCACTTTGGA	IVS1+1405 to IVS1+1424	
G	TGTCAAAACTCTCGATCTCAGG	IVS1+1363 to IVS1+1384	386
	TGGGCAACAGAGTAAGACTTCA	IVS1+1727 to IVS1+1748	
H	ACAGGGGCTCATGAGAAATG	IVS1+1659 to IVS1+1678	384
	CCCACACAGACAATTCTTTATTCA	IVS1+2019 to IVS1+2042	
I	CGTGCCCAGCCTATTATCTT	IVS1+1994 to IVS1+2013	386
	GAGACCAGCCTGACCAACAT	IVS1+2340 to IVS1+2379	
J	CCATGCCTGGCTAATTTTGT	IVS1+2314 to IVS1+2333	365
	TTTTGGCAGATGTCTCTTCTCA	IVS1+2657 to IVS1+2678	

* IVS, intervening sequence.

## Results

### Screening of colorectal and gastric cancer tissue samples with MLH1 promoter methylation

**Figure 2:**
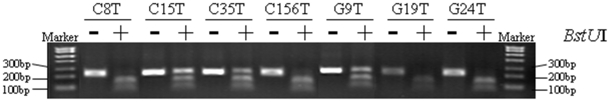
MLH1 promoter methylation assessment by COBRA with *BstU*I. The four colorectal cancers (C8T, C15T, C35T and C156T) and 3 gastric cancers (G9T, G19T and G24T) were analysed. Symbol “−” means that PCR products were digested without *BstUI*, and “+” means digestion with *BstUI*.

Of the 188 colorectal and 27 gastric cancer tissues, 48 colorectal and 9 gastric cancer samples were found to have MSI positive phenotype. The MSI positive samples were then examined for MLH1 promoter methylation, and 4 colorectal and 3 gastric cancer samples exhibited MLH1 promoter methylation (Fig. 2).

### Association of MLH1 methylation and negative or weak expression of MLH1

**Figure 3:**
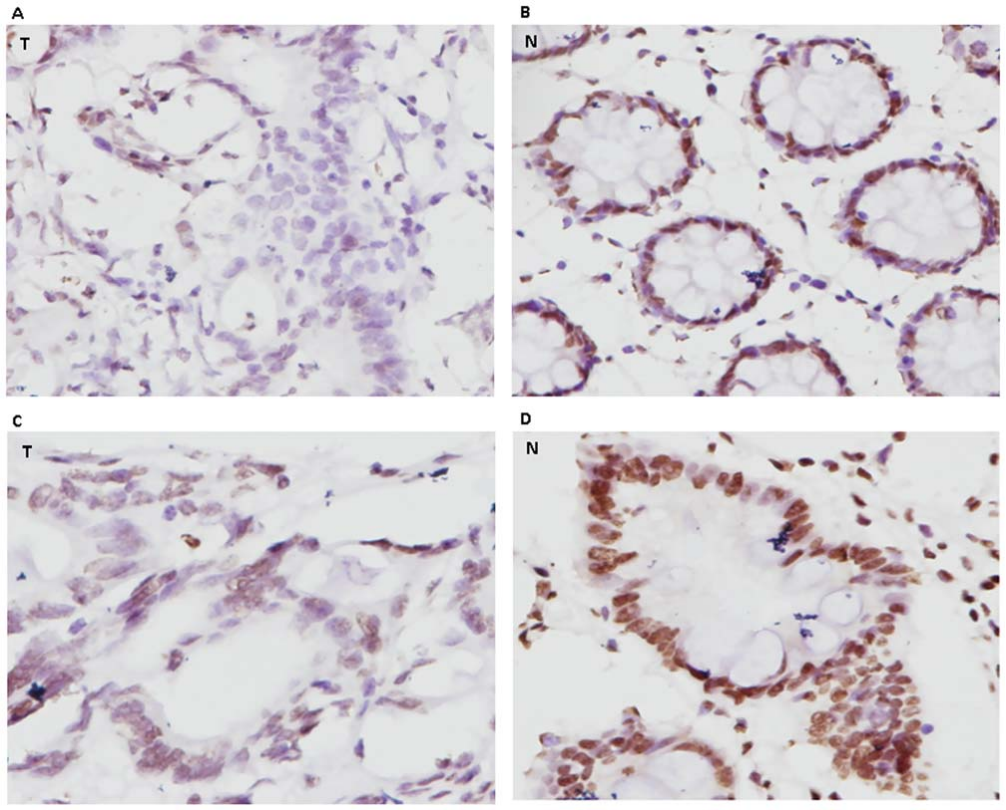
IHC staining of MLH1 expression in tumours and their adjacent normal tissues. A and B represent a colorectal tumour (T) and the adjacent normal tissue (N) respectively; C and D display a gastric tumour (T) and the adjacent normal tissue (N). A: negative staining; C: weak staining; B and D: strong staining.

The seven MLH1 methylation samples, together with their adjacent normal tissues, were analysed for MLH1 expression using IHC staining. All seven cancer tissues showed negative or weakly positive expression of Mlh1, and their adjacent normal tissues had strong expression (Fig. 3).

### Comparison of MLH1 methylation patterns between normals and cancers

**Figure 4:**
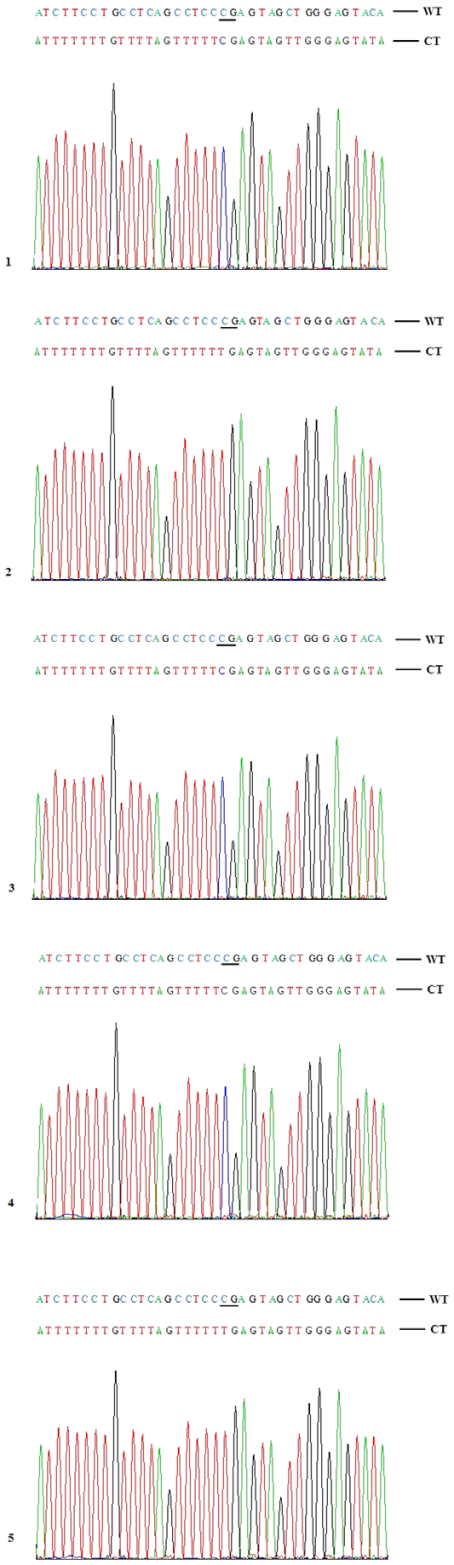
Partial sequencing data for 5 clones of an PCR amplicon located at 116+1780 to 116+2059 of MLH1 in RKO cells. The CpG sites are underscored. Clones 1, 3 and 4 displayed methylation, and clones 2 and 5 no methylation. The methylation frequency of the CpG site is 60%. WT, wildtype; CT, converted type.

**Figure 5:**
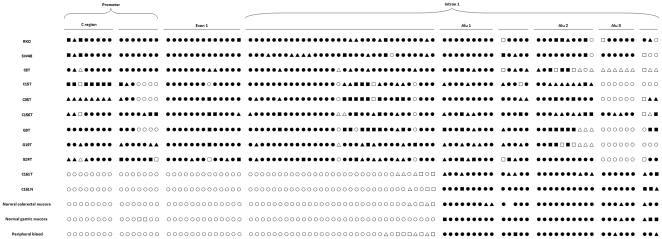
Methylation profiles of CpG sites of the *MLH1* C region containing promoter, exon 1 and majority of intron 1 in normal colorectal and gastric mucosa and peripheral blood, colorectal cancer cells and tissues, and gastric cancer tissues. In total 93 CpG sites are analysed. However, 92 CpG sites can be seen in the normal colorectal mucosa, due to a transition of G to A at a CpG site between Alus 1 and 2. •,▴,▪,□,△ and ○ represent methylation frequency of 100%, 80%, 60%, 40%, 20% and 0, respectively. 80% indicates that of the 5 clones, 4 exhibited methylation at a CpG site. RKO and SW48: colorectal cells; C8T, C15T, C35T, C156T: MSI positive colorectal cancer tissues; C161T: MSI negative colorectal cancer; C161N: C161T matched normal tissue; and G9T, G19T and G24T: MSI positive gastric cancers.

A total of 93 CpG sites located on the region analysed were measured for methylation status using bisulfite genomic sequencing (Fig. 4). The two colorectal cancer cell lines, four MSI positve colorectal cancer tissues, and three MSI positive gastric cancer tissues showed different patterns compared to the MSI negative colorectal cancer tissue, the normal colorectal and gastric mucosa and peripheral blood (Fig. 5). The MSI negative colorectal cancer tissue, the normal colorectal and gastric mucosa and peripheral blood displayed no methylation in the *MLH1* promoter, and demethylation or hypomethylation (less than 50%) in the regions between promoters and Alu elements, whereas the regions within or downstream of Alu elements exhibited hypermethylation (more than 50%). In contrast, for the colorectal cancer cells and tissues (MSI positive) and gastric cancer tissues (MSI positive), the *MLH1* promoters and the regions between promoters and Alu elements are hypermethylated, with the exception of a very few hypomethylated CpG sites. However, the regions within or downstream of Alu elements showed a certain degree of hypomethylation or demethylation compared to the normal tissues. Furthermore, the hypomethylation and demethylation were more frequently seen in the colorectal and gastric cancer tissues (MSI positive) compared to the colorectal cancer cells (Fig. 5).

### Mutation screening in the cancer cells and tissues

**Figure 6:**
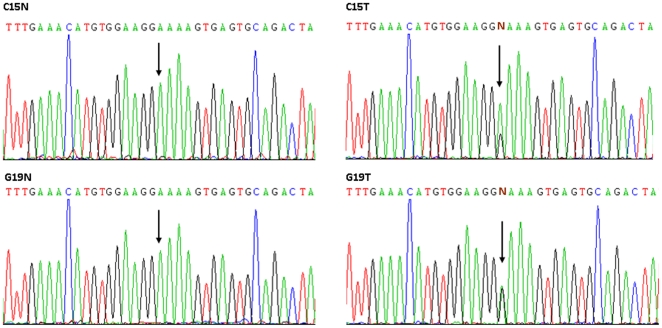
Sequencing analysis of MLH1 5′ end. One colorectal and one gastric cancer, C15T and G19T, exhibited an identical heterozygous mutation, A→G, at the same site, IVS1 +681 bp. Their adjacent normal tissues, C15N and G19N, did not show the mutation. Arrows display the sites of the mutations.

Mutations in the *MLH1 5′* end for methylation analysis were screened in the 2 cancer cells and 7 cancer tissues. One colorectal and one gastric cancer tissue were found to have mutations compared to their adjacent normal tissues (Fig. 6). One colorectal and one gastric cancer exhibited an identical heterozygous mutation, A→G, at the same site, IVS1 +681 bp. Their adjacent normal tissues did not show the mutation, indicating that it is somatic and tumour-specific.

## Discussion

The methylation patterns of Alu elements in the *MLH1* have not previously been explored in normal tissues. In this study, we analysed the methylation status of the CpG sites within the three Alu elements located in the intron 1 of *MLH1*, and found that all three Alu elements are at levels of hypermethylation in normal colorectal and gastric mucosa and peripheral blood (Fig. 5). It is in line with earlier finding that Alu elements are hypermethylated in the normal gastric mucosa, breast epithelial and kidney tissues [6], [8]. The MSI negative colorectal tumour displayed very similar methylation pattern to the normal tissues (Fig. 5). Normally, methylation within the Alu elements should not spread to the adjacent CpG islands due to blockage by the Sp1 elements [9], [10]. Here we revealed that the regions upstream of the Alu elements are unmethylated or hypomethylated in the normal colorectal and gastric mucosa and peripheral blood. Clear boundaries are seen between hypermethylated Alu elements and hypomethylated regions upstream of them (Fig. 5). There are several hypomethylated CpG sites upstream of the Alu elements in the normal tissues, and those CpG sites are out of the C region (Fig. 5), suggesting a limited spread of Alu methylation in normal cells. Some CpG sites around or within the Alu elements in normal tissues displayed less than 100% methylation (Fig. 5), suggesting that methylation of individual sites is not clonally derived, even though the overall pattern of methylation is transmitted from cell to cell. In line with this, it has been shown that the mouse adenine phosphoribosyltransferase gene has methylation patterns which differ between liver cells [10]. The mechanism behind this could be that the methylation centres of the identical genes in different cells have variable strength of signal travelling upstream or downstream [11].

Disruption of the Sp1 elements facilitates *de novo* methylation of the adjacent CpG sites [9], [10], [12], and induces epigenetic gene inactivation [12]. It is proposed that Alu elements are the methylation centres in the genome [8]. The methylation patterns of the Alu elements in the *MLH1* methylated colorectal cancer cells RKO and SW48 have not been studied previously. RKO and SW48 have been found to have methylation in the *MLH1* promoter region, particularly in the C region (−310 to −240, relative to the initiation codon), which causes reduced *MLH1* expression [2]. In this study we also analysed methylation patterns in the *MLH1* promoters, the three Alu elements and the regions between promoters and the Alu elements. Compared to the normal colorectal and gastric mucosa and peripheral blood, the Alu elements showed hypomethylation especially in Alus 2 and 3 in both RKO and SW48. Moreover, the region downstream of Alu 3 showed hypomethylation in both RKO and SW48 (Fig. 5). However, the promoters and particularly the regions between promoters and Alu elements were hypermethylated in RKO and SW48 (Fig. 5). This strongly suggests that the *MLH1* promoter methylation spreads from the methylation centres within intron 1 of *MLH1*.

Because cultured cancer cell lines are considered to have a higher degree of methylation than primary tumours [13], we decided to analyse the primary tumours for MLH1 methylation pattern in the same regions as the cell lines. A somewhat lower degree of methylation was detected in the MLH1 promoter region in the 4 MSI positive colorectal and 3 MSI positive gastric cancers compared to the two cell lines (Fig. 5). However, hypermethylated promoters, particularly in the C regions, and the regions between promoters and Alu elements are seen in all seven cancers analysed. Some regions within and around Alu elements were obviously hypomethylated in the tumour tissues. Hence the data from the cancer tissues strongly suggest that the methylation is spreading from the Alu elements to the 5′ region of the MLH1 in MSI positive colorectal and gastric cancer tissues, as well as in the colorectal cancer cell lines. Alu hypomethylation has been identified in gastric carcinomas and melanoma cell lines [6], [14]. Another repetitive sequence, LINE-1, also showed hypomethylation in malignant gastrointestinal stromal tumours [15]. Hypomethylation may increase the malignant potential of tumours by inducing accumulation of chromosomal aberrations or methylation spreading to the promoters of tumour suppressor genes. Thus methylation of the repetitive sequences may be a useful marker for malignancy assessment.

A Sp1 element was identified at −119 of the *MLH1* promoter region using transcription factor search software (TFSEARCH ver.1.3). To ascertain whether there are mutations in the Sp1 element, we sequenced the entire region analysed above in the cancer cell lines and tissues. No mutations in the Sp1 element were detected, but one MSI positive colorectal and one MSI positive gastric cancer showed the A→G mutation, at the same site, IVS1 +681 bp in the MLH1 gene. This site is between the promoter and Alu elements. Our finding suggests that the region is a mutational hotspot in the pathogenesis of colorectal and gastric cancer. We speculate that the *trans-acting* elements binding to this site, or other sites which are between promoter and Alu elements, may be involved in the methylation spreading from the Alu elements to the promoter region. Therefore, those *trans-acting* elements could behave as guardians of the methylation centres, e.g. Alu elements. Further studies are needed in order to search for those *trans-acting* elements, which could be the therapeutic targets of cancers in the future.

## Supporting Information

Table S1 **Oligonucleotide sequences of the primers for methylation analysis.**
(DOC)

## References

[1] Goodfellow PJ, Buttin BM, Herzog TJ, Rader JS, Gibb RK (2003). Prevalence of defective DNA mismatch repair and MSH6 mutation in an unselected series of endometrial cancers.. Proc Natl Acad Sci U S A.

[2] Deng G, Chen A, Hong J, Chae HS, Kim YS (1999). Methylation of CpG in a small region of the h*MLH1* promoter invariably correlates with the absence of gene expression.. Cancer Res.

[3] Kang GH, Lee S, Shim YH, Kim JC, Ro JY (2002). Profile of methylated CpG sites of h*MLH1* promoter in primary gastric carcinoma with microsatellite instability.. Pathol Int.

[4] Chen H, Taylor NP, Sotamaa KM, Mutch DG, Powell MA (2007). Evidence for heritable predisposition to epigenetic silencing of *MLH1*.. Int J Cancer.

[5] Mei M, Liu D, Dong S, Ingvarsson S, Goodfellow PJ (2010). The *MLH1* -93 promoter variant influences gene expression.. Cancer Epidemiol.

[6] Xiang S, Liu Z, Zhang B, Zhou J, Zhu BD (2010). Methylation status of individual CpG sites within Alu elements in the human genome and Alu hypomethylation in gastric carcinomas.. BMC Cancer.

[7] Turker MS, Bestor TH (1997). Formation of methylation patterns in the mammalian genome.. Mutat Res.

[8] Graff JR, Herman JG, Myöhänen S, Baylin SB, Vertino PM (1997). Mapping patterns of CpG island methylation in normal and neoplastic cells implicates both upstream and downstream regions in de novo methylation.. J Biol Chem.

[9] Brandeis M, Frank D, Keshet I, Siegfried Z, Mendelsohn M (1994). Sp1 elements protect a CpG island from de novo methylation.. Nature.

[10] Macleod D, Charlton J, Mullins J, Bird AP (1994). Sp1 sites in the mouse aprt gene promoter are required to prevent methylation of the CpG island.. Genes Dev.

[11] Mummaneni P, Bishop PL, Turker MS (1993). A cis-acting element accounts for a conserved methylation pattern upstream of the mouse adenine phosphoribosyltransferase gene.. J Biol Chem.

[12] Mummaneni P, Walker KA, Bishop PL, Turker MS (1995). Epigenetic gene inactivation induced by a cis-acting methylation center.. J Biol Chem.

[13] Smiraglia DJ, Rush LJ, Frühwald MC, Dai Z, Held WA (2001). Excessive CpG island hypermethylation in cancer cell lines versus primary human malignancies.. Hum Mol Genet.

[14] Tellez CS, Shen L, Estécio MR, Jelinek J, Gershenwald JE (2009). CpG island methylation profiling in human melanoma cell lines.. Melanoma Res.

[15] Igarashi S, Suzuki H, Niinuma T, Shimizu H, Nojima M (2010). A novel correlation between LINE-1 hypomethylation and the malignancy of gastrointestinal stromal tumors.. Clin Cancer Res.

