# Supplementary material for: Spreading of Alu Methylation to the Promoter of the MLH1 Gene in Gastrointestinal Cancer
Source: PLoS One. 2011 Oct 12;6(10):e25913. doi: 10.1371/journal.pone.0025913 (PMC3192117; doi:10.1371/journal.pone.0025913)
Supplement: Table S1 — Oligonucleotide sequences of the primers for methylation analysis. (DOC) [file pone.0025913.s001.doc]

Table S1. Oligonucleotide sequences of the primers for methylation analysis

| Primer name | Primer sequence (5’-3’) | Genomic position | Product size (bp) |
| --- | --- | --- | --- |
| 1 | GTTAAGTATTTTTTTCGTTTTGCGT | -400 to -375 | 226 |
|  | CTCTATAAATTACTAAATCTCTTCGTC | -201 to -175 |  |
| 2 | GGTATTTTTGTTTTTATTGGTTGGATAT | -339 to -312 | 193 |
|  | CAACTTAAATACCAATCAAATTTCTCA | -173 to -147 |  |
| 3 | GGTAGTAGTCGTTTTAGGGAGGGAC | -223 to -199 | 204 |
|  | AAAAAACGTCTAAATACTCAACGAA | -44 to -20 |  |
| 4 | TAGTAGTTGTTTTAGGGAGGGATGA | -221 to -197 | 204 |
|  | CAAAAAAACATCTAAATACTCAACAAA | -44 to -18 |  |
| 5 | AGAGTTGAGAAATTTGATTGGT | -178 to -157 | 295 |
|  | CCAATTCTCAATCATCTCTTT | +97 to IVS1+1 |  |
| 6 | GTTTTTTTGGCGTTAAAATGTC | -17 to +5 | 242 |
|  | ATACGCTATACATACCTCTACCCGA | IVS1+85 to IVS1+109 |  |
| 7 | TTTTTGGCGTTAAAATGTCGTTC | -14 to +9 | 168 |
|  | GTAACCCTTAAATAAACCCGACTCG | IVS1+14 to IVS1+38 |  |
| 8 | TTTTTGGTGTTAAAATGTTGTTTGT | -14 to +11 | 171 |
|  | ATCATAACCCTTAAATAAACCCAACTCA | IVS1+14 to IVS1+41 |  |
| 9 | AAAGAGATGATTGAGAATTGGT | +97 to IVS1+2 | 384 |
|  | AAAATTTTTCTCCCCATCTT | IVS1+345 to IVS1+364 |  |
| 10 | GTATGTTTATAACGGCGGAGGTCGT | IVS1+106 to IVS1+130 | 228 |
|  | CATCTTTTAACTTCGCATATTCTACATACA | IVS1+304 to IVS1+333 |  |
| 11 | TGTTTGGAGTGTAAGTGGAGGA | IVS1+218 to IVS1+239 | 294 |
|  | TCAAACCCAAAATACATCAACC | IVS1+490 to IVS1+511 |  |
| 12 | TTTATTTTGAGTTTTTTTTAAAATAAAT | IVS1+443 to IVS1+470 | 171 |
|  | AAATAACCCATAAAATCTAATATCTTC | IVS1+587 to IVS1+613 |  |
| 13 | TATTTTGAGTTTTTTTTAAAATAAATCGT | IVS1+445 to IVS1+473 | 168 |
|  | AATAACCCATAAAATCTAATATCTTCGAA | IVS1+584 to IVS1+612 |  |
| 14 | GGTTGATGTATTTTGGGTTTGA | IVS1+490 to IVS1+511 | 344 |
|  | CCCTCTAAAAACCTACCACTCC | IVS1+812 to IVS1+833 |  |
| 15 | TCGGAGGACGTATTTCGAAGATATTA | IVS1+571 to IVS1+596 | 216 |
|  | AACGTACGAACGTCACTAACAACTAA | IVS1+761 to IVS1+786 |  |
| 16 | TTTTGTTAAAATTAGTTGTTAGTGA | IVS1+750 to IVS1+774 | 318 |
|  | CAATACAATAATAAACCCAATTATCC | IVS1+1042 to IVS1+1067 |  |
| 17 | GGAAGGTAGATGAAAGAAGGAG | IVS1+921 to IVS1+942 | 285 |
|  | CCACTACACTCCAACCTAAATAACAA | IVS1+1180 to IVS1+1205 |  |
| 18 | AAATATATTAGTTTGGTGGTTTGTAATTAA | IVS1+1093 to IVS1+1122 | 264 |
|  | TAACCAACATAAAAAAATCCCATCT | IVS1+1332 to IVS1+1356 |  |
| 19 | ATGTTGGTTAGGTTGGTGTTAAAAT | IVS1+1347 to IVS1+1371 | 181 |
|  | ACAAATCACCTAAAATCAAAAAATC | IVS1+1503 to IVS1+1527 |  |
| 20 | ATGTTGGTTAGGTTGGTGTTAAAAT | IVS1+1347 to IVS1+1371 | 445 |
|  | CAAAAATTACCCTAAACCAAAATCA | IVS1+1767 to IVS1+1791 |  |
| 21 | TTTTTTAAAGTGTTGGGATTATAGG | IVS1+1402 to IVS1+1426 | 258 |
|  | AAACTCCATATAAAAATCTCCACTTAAACC | IVS1+1579 to IVS1+1608 |  |
| 22 | TGGTTTTTTAAAGTGTTGGGATTATA | IVS1+1534 to IVS1+1559 | 207 |
|  | CAAAAATTACCCTAAACCAAAATCA | IVS1+1767 to IVS1+1791 |  |
| 23 | TAGGGGTTTATGAGAAATGTTT | IVS1+1660 to IVS1+1681 | 253 |
|  | ACCAACATAAAAAAACCCC | IVS1+1894 to IVS1+1912 |  |
| 24 | GGGTAATTTTTGTTTTTTGGTTTTAAG | IVS1+1780 to IVS1+1806 | 280 |
|  | AAAAAAATTATCTATTCCCCACACAAAC | IVS1+2032 to IVS1+2059 |  |
| 25 | TTGGTTTGTGTTTATAGGTTATTTTT | IVS1+2099 to IVS1+2124 | 462 |
|  | AAATAATTTTCTCTACTATTCCCTTTT | IVS1+2534 to IVS1+2560 |  |
| 26 | TGGTTAGGTTGGTTTTAAATTTTTG | IVS1+2364 to IVS1+2388 | 379 |
|  | AAAACATTCAACACTATTTCTATTCCTC | IVS1+2715 to IVS1+2742 |  |
| 27 | GTTTGGTTAGAAAATTTATTGAT | IVS1+2452 to IVS1+2474 | 425 |
|  | AACAAACTCCAAATACAAACAA | IVS1+2855 to IVS1+2876 |  |

* IVS, intervening sequence; 1, 3/4, 6, 7/8, 10, 13 and 15, methylation-specific PCR (MSP) primers.
